# Determinants of circulating microRNA‐125b, a risk predictor of vascular calcification, among community‐dwelling older adults^*^

**DOI:** 10.1002/ctm2.145

**Published:** 2020-08-12

**Authors:** Chia‐Ter Chao, Hsiang‐Yuan Yeh, Der‐Sheng Han, Jenq‐Wen Huang, Kuo‐Chin Huang

**Affiliations:** ^1^ Nephrology Division Department of Internal Medicine National Taiwan University Hospital BeiHu Branch Taipei Taiwan; ^2^ Graduate Institute of Toxicology National Taiwan University College of Medicine Taipei Taiwan; ^3^ Geriatric and Community Medicine Research Center National Taiwan University Hospital BeiHu Branch Taipei Taiwan; ^4^ School of Big Data Soochow University Taipei Taiwan; ^5^ Department of Rehabilitation and Physical Medicine National Taiwan University Hospital BeiHu Branch Taipei Taiwan; ^6^ Nephrology Division Department of Internal Medicine National Taiwan University Hospital Yunlin Branch Yunlin County Taiwan; ^7^ Department of Family Medicine National Taiwan University Hospital BeiHu Branch Taipei Taiwan; ^8^ Department of Family Medicine National Taiwan University Hospital Taipei Taiwan; ^9^ Department of Family Medicine College of Medicine National Taiwan University Taipei Taiwan

**Keywords:** aortic calcification, biomarker, diabetes mellitus, epigenetics, geriatrics, hemoglobin, microRNA, obesity, red cell distribution width, vascular calcification

Dear editor,

Vascular calcification (VC) describes the ectopic calcium deposition within vascular walls among those with diabetes mellitus (DM), chronic kidney disease (CKD), and those of advanced age.^1^ The current understandings of VC pathophysiology are complex,^2^ and vascular ageing is an important contributor;^3^ this is reflected in the rising VC incidence in older adults and the associated cardiovascular risk in this age group. From the cellular perspective, senescence of vascular smooth muscle cells (VSMCs) serves as a critical player during VC; the replicative senescence of VSMCs or endothelial cells lead to experimental VC. In this sense, the process of biological aging is intimately linked to VC susceptibility.

Existing literature dictates that multiple macromolecule‐based biomarkers of VC are available. There has been a growing interest in harnessing epigenetic biomarkers for the diagnosis of VC, especially microRNA (miR)‐based ones. However, circulating miRNA levels tend to decline with worsening renal function, though their clinical utility seems unaffected. Multiple miRNAs have been shown to play a role in uremic VC, including miR‐26a, miR‐29 and miR‐34 family, miR‐133, miR‐223, etc.^2^ miRNAs influence a given individual's susceptibility to VC, enhance or suppress VC progression, and potentially serves as indicators of treatment responses regarding VC management. Among these VC‐related miRNAs, miR‐125b is an important example; circulating miR‐125b levels reportedly predict the clinical course of VC in patients with CKD.^4^, ^5^ Judging from the importance of miR‐125b in vascular pathologies shown by a prior review^6^ and its readiness for assay, measuring circulating miR‐125b levels for vascular risk estimation may bear high clinical utility. However, determinants of circulating miR‐125b levels remain unclear. We harnessed a prospectively enrolled cohort of relatively healthy older adults for examining the clinical and laboratory determinants of their circulating miR‐125b levels. The details of the cohort establishment procedure, plasma miRNA measurement and quantification, and statistical analysis strategies are listed in the Supporting information.

A total of 384 community‐dwelling older adults were consecutively enrolled during 2017, with a mean age of 74.1 ± 6.2 years (Supporting information Table S1). Enrollees kept a healthy lifestyle with few smokers (3.1%) or regular drinkers (22.1%). Less than half had comorbidities and they took few medications. Their physical examination parameters and laboratory data are shown in Supporting information Table S1.

The mean and median values of circulating miR‐125b among enrollees were 0.0783 ± 0.3144 and 0.0116 (0.0031, 0.0392), respectively. The Kolmogorov‐Smirnov test *P* value for miR‐125b levels was < .001, suggesting that its distribution was nonparametric. We normalized circulating miR‐125b levels using logarithmic transformation, and the distribution now became normalized (Figure 1A) with a mean value of −1.9482 ± −0.8287 and a Kolmogorov–Smirnov *P* value of .2. We subsequently divided enrollees into those with a high (n = 186; 48.4%) and low (n = 198; 51.6%) circulating miR‐125b levels based on the median value. Those with a high miR‐125b were more likely to be male (*P *= .03), less likely to have DM (*P* = .016) and use antidiabetic medications (*P* = .02), had a significantly higher height (*P* = .041), weight (*P *= .048), and waist circumference (WC) (*P* = .049), a higher hemoglobin (*P* = .012) and a lower red‐cell distribution width (RDW) (*P* = .037) than those with a low miR‐125b (Supporting information Table S1). Similarly, enrollees with DM and received antidiabetic medications had significantly lower log‐transformed circulating miR‐125b levels than those without (*P* = .003 and .003, respectively) (Supporting information Table S2, with the nontransformed data shown in Supporting information Table S3). Correlation analyses revealed that log‐transformed miR‐125b negatively correlated with RDW levels (*P* = .03) (Figure 1B) but not with other laboratory parameters.

**FIGURE 1:**
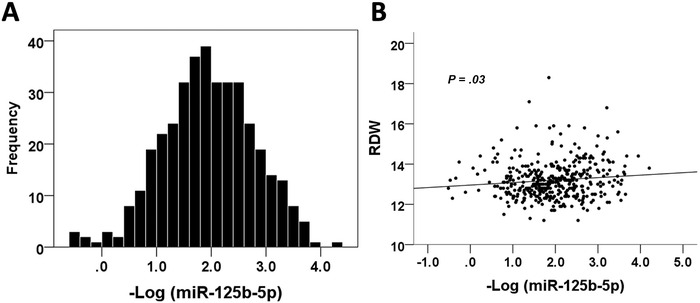
(A) Frequency distribution plot of circulating miR‐125b levels among study participants. (B) The correlation plot between minus log‐transformed miR‐125b level and RDW levels. RDW, red cell distribution width

Multiple regression analyses with having a high circulating miR‐125b level as the dependent variable were done, incorporating variables with significant differences in univariate analysis (Supporting information Table S4). Model 1 included age, gender, DM, the use of antidiabetic medications, height, weight, and WC, while model 2 included model 1 variables, hemoglobin, and RDW. In model 1, having DM was significantly associated with a lower probability of having a high circulating miR‐125b (odds ratio [OR] 0.362, 95% confidence interval [CI] 0.177‐0.738), while higher WC was associated with an increased probability (OR: 1.029, 95% CI 1.006‐1.052) (Supporting information Table S4). In model 2, the relationship between DM, WC, and a high circulating miR‐125b remained significant, while higher RDW showed an inverse association with miR‐125b (OR: 0.78, 95% CI: 0.617‐0.986) (Supporting information Table S4). There was no collinearity between variables observed in the regression models (variance inflation factors for all variables < 5).

In this study, we showed that having DM was independently associated with a lower circulating miR‐125b level, while a higher WC and lower RDW was associated with a higher circulating miR‐125b level among healthy older adults. Based on these findings, it would be prudent to adjust for these variables in subsequent studies investigating the clinical utility of this epigenetic VC biomarker.^7^

We identified that DM, increased WC or central obesity, and a higher RDW significantly influenced circulating miR‐125b levels (Supporting information Table S4). Plausible reasons for this phenomenon can be multiple; diabetic mice exhibited diminished miR‐125b expressions within β‐cells, and miR‐125b upregulation restored insulin sensitivity.^8^ It is possible that circulating miRNA levels resonate with tissue‐specific expression status, particularly in patients with chronic diseases such as DM. Similarly, an increased WC often coexist with a greater amount of central adipose tissues, and the expressions of miR‐125b are reportedly upregulated during adipogenesis^9^; furthermore, miR‐125b could be detectable in extracellular vesicles released from adipogenic mesenchymal stem cells,^9^ suggesting that adipocytes might be a potential source of miR‐125b. Finally, endothelial cells and plausibly VSMCs can release extracellular vesicles containing miR‐125b,^4^ exerting paracrine influences on vascular pathologies. Higher RDW is frequently driven by occult inflammation, an occult modulator of miRNA expressions in vascular cells.^10^ These evidences support the notion that they modify circulating miR‐125b levels through altering their expressions in different tissues (Figure 2).

**FIGURE 2:**
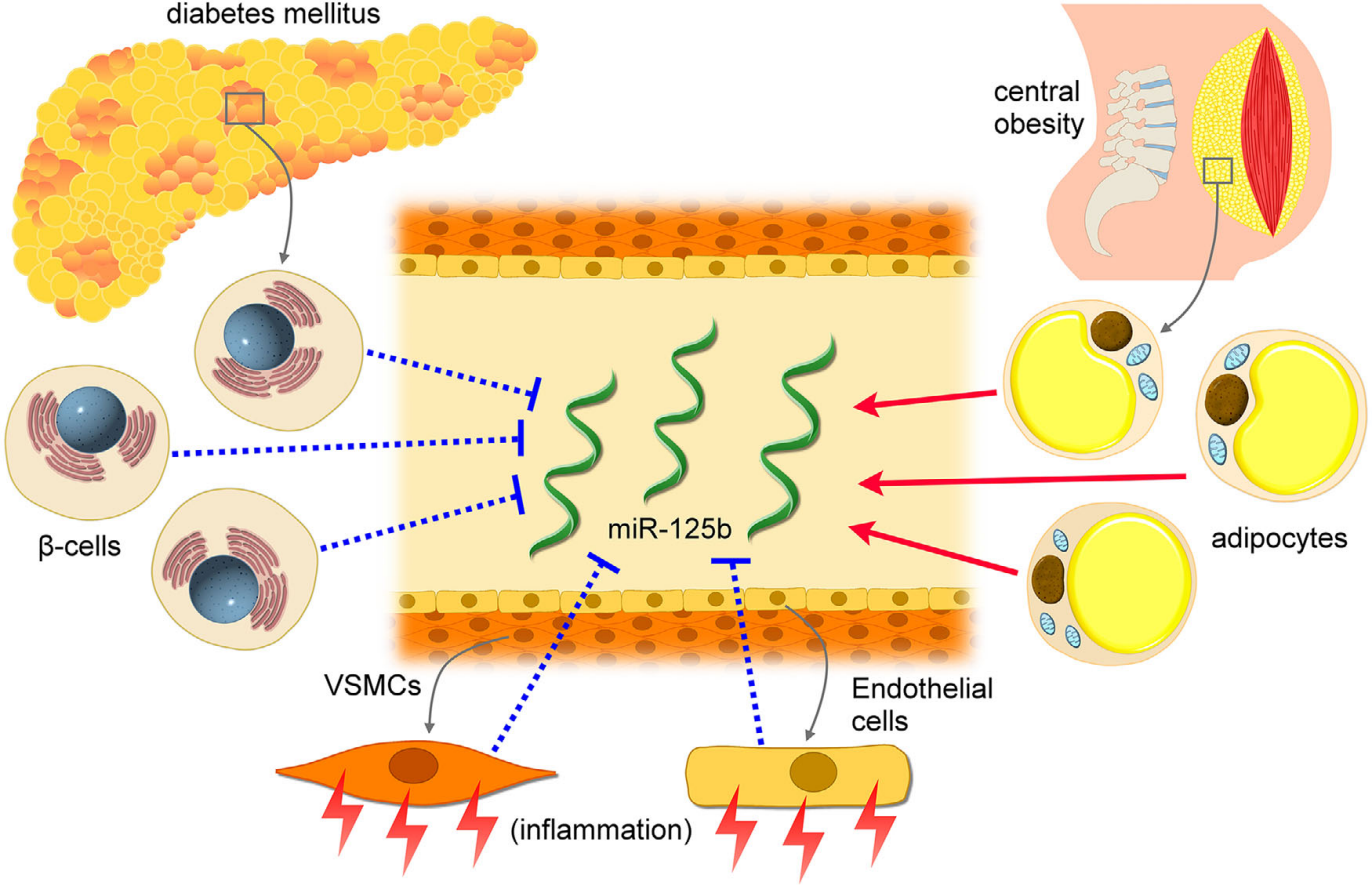
A putative diagram illustrating the potential mechanisms through which independent factors affect circulating miR‐125b levels. VSMC, vascular smooth muscle cell

miR‐125b have been repeatedly shown to be pathogenetically important in vascular remodeling during CKD, especially VC. Being termed a vascular miRNA, miR‐125b reportedly drives the phenotypic switch of contractile VSMCs to synthetic ones, via regulating the expressions of Ets‐1, osterix, and RUNX2.^6^ This effect results in the osteogenic differentiation of VSMCs and leads to the propagation of VC; such process is further augmented by the adverse influences introduced by uremic milieu including proinflammatory cytokines, uremic toxins, and aberrant divalent ion levels.

Our study has its limitations. We did not measure other markers potentially associated with VC such as Klotho, C‐reactive protein, and oxidative stress. Nonetheless, we believe that these factors are unlikely to influence our findings significantly, since the vascular burden among our participants was expectedly low. Further mechanistic and clinical applicability studies are needed to extend our findings.

In conclusion, from a moderate‐sized cohort of prospectively enrolled healthy older adults, we discovered that DM and RDW were inversely associated with circulating miR‐125b levels, while WC exhibited a positive association. These findings facilitate the accurate analysis of circulating miR‐125b regarding its clinical utility, and indirectly suggest that miR‐125b may be one of the mediators between DM and the risk of VC in older adults.

## ETHICS, CONSENT, AND PERMISSION

The protocol of the current study was approved by the Institutional Review Board of the National Taiwan University Hospital (NO. 201601091RIND). Written informed consent has been obtained from all participants.

## CONSENT FOR PUBLICATION

Not applicable.

## AVAILABILITY OF DATA AND MATERIAL

The raw data for conducting this analysis are available upon reasonable request to the corresponding author.

## COMPETING INTERESTS

The authors have no relevant financial or nonfinancial competing interests to declare in relation to this manuscript.

## FUNDING DISCLOSURE

The study is financially sponsored by National Taiwan University Hospital BeiHu Branch and Ministry of Science and Technology, Taiwan (MOST 108‐2314‐B‐002‐055‐ and MOST 109‐2314‐B‐002‐193‐MY3).

## AUTHOR CONTRIBUTIONS

Study design: CTC, DSH; Data analysis: CTC, HYY, JWH; Article drafting: CTC, HYY, DSH, JWH, KCH; All authors approved the final version of the manuscript.

## SPONSOR'S ROLE

The sponsors have no role in the study design, data collection, analysis, and result interpretation of this study.

## Supporting information

Supporting Information
