# Supplementary material for: Determinants of circulating microRNA‐125b, a risk predictor of vascular calcification, among community‐dwelling older adults*
Source: Clin Transl Med. 2020 Aug 12;10(4):e145. doi: 10.1002/ctm2.145 (PMC7423181; doi:10.1002/ctm2.145)
Supplement: Supplementary file 1 — Supporting Information [file CTM2-10-e145-s001.doc]

**Methods**

*Ethical statement*

The protocol of the current study adhered to the Declaration of Helsinki, and has been approved by National Taiwan University Hospital (NO. 201601091RIND). All participants provided written informed consent prior to enrollment.

*Establishment of the study cohort*

Community-dwelling older adults (defined as those ≥ 65 years) were prospectively recruited from the out-patient clinics of National Taiwan University Hospital BeiHu Branch, geriatric health examination program, and long-term care service centers from the Taipei City in 2017. Exclusion criteria consisted of those who could not communicate due to dementia or consciousness disturbance. After participants signed the informed consent, we documented their demographic profiles, lifestyle factors, and comorbidities across multiple organ systems including cardiovascular, pulmonary, gastrohepatic, endocrinologic, kidney systems, and malignancies. Their regular medication regimens were also ascertained. Participants then underwent physical assessment, with anthropometric parameters (body height [BH], body weight [BW], body mass index [BMI], and waist circumference [WC]), blood pressure (BP) and heart rate (HR) recorded. Participants were then instructed to receive blood drawing in the morning at a fasting state, before they took their regular medications. Part of the specimens was sent for hemogram and biochemical examinations including nutritional profiles, renal function tests, and metabolic panels), while others were cryopreserved for circulating microRNA analysis.

*Measurement of circulating miR-125b levels*

The procedures for measuring miR-125b levels from plasma has been detailed previously [1,2]. We used the cryopreserved plasma from participants, as the stability of miRNA in such storage condition and during freezing-thawing has been shown to be excellent [3]. In brief, we used the miRNeasy Serum/Plasma kit (QIAGEN, Netherland) to extract cell-free total RNAs from cryopreserved plasma of small volume, according to the manufacturer’s protocol; after adding in a fixed amount of spike-in control (*Caenorhabditis elegans* miR-39; QIAGEN, Netherland), plasma were subject to spin columns for small RNA extraction and purifications, followed by elution and centrifugation. Purified small RNAs were subsequently processed through reverse transcription (miScript RT kit; QIAGEN, Netherland) and polymerase chain reaction for quantitation purpose. Each sample was done in technical triplicates, with the averages calculated. Inter-plate variations were mitigated by using internal controls in each plate. The utilization of *C. elegans* miR-39 as an extraction quality control has been shown to optimize RNA yields and reduce variability in results, allowing for cross-study comparison [4]. For the reference group, we used plasma from healthy young individuals (age 20 to 30 years) without any underlying diseases. ΔΔCt method was used for calculating circulating miR-125b levels.

*Statistical analysis*

In this study, we described continuous variables in means ± standard deviations (if parametric) or medians with interquartile ranges (if non-parametric), while categorical variables were expressed in numbers with percentages in parentheses. For comparisons between 2 groups of parametric and non-parametric continuous variables, we used the independent *t*-test and Mann-Whitney *U* test, respectively, while the chi-square test was used to compare categorical variables. One-way analysis of variance was utilized for comparing variables of > 2 groups.

We first examined the distribution of circulating miR-125b levels using the Kolmogorov-Smirnov test. A leftward skewed distribution was detected, and we normalized miR-125b levels using logarithmic transformation, whose values were used in the subsequent analyses. For analytic purpose, we divided the participants based on the median value into two groups, namely, the high and low circulating miR-125b ones. Demographic features, lifestyle factors, comorbidities, medication regimens, physical parameters, and laboratory profiles were compared between older adults with high and low circulating miR-125b levels. We further compared log-transformed miR-125b levels based on categorical variables. We also examined the correlation between log-transformed miR-125b levels and continuous variables from the recorded clinical characteristics, using the Pearson’s correlation coefficient. This is followed by multiple regression analyses with stepwise backward variable selection, using high circulating miR-125b level as the dependent variable, incorporating all significant variables in the univariate analysis and those exhibiting significant correlation with miR-125b. Two sets of regression analyses were arranged; the first one included clinical and physical parameters, while the second one additionally included laboratory data. Issues of collinearity were examined using the variance inflation factor (VIF) approach. We used SPSS version 19.0 in all analyses, and a *p* value less than 0.05 was considered statistically significant.

**Reference**

1. Chao C-T, Yuan T-H, Yeh H-Y et al (2019) Risk Factors Associated With Altered Circulating Micro RNA -125b and Their Influences on Uremic Vascular Calcification Among Patients With End-Stage Renal Disease. J Am Heart Assoc 8:e010805.

2. Chao C-T, Liu Y-P, Su S-F et al (2017) Circulating MicroRNA-125b Predicts the Presence and Progression of Uremic Vascular Calcification. Arterioscler Thromb Vasc Biol 37:1402-1414.

3. Rounge TB, Lauritzen M, Langseth H et al (2015) microRNA Biomarker Discovery and High-Throughput DNA Sequencing Are Possible Using Long-term Archived Serum Samples. Cancer Epidemiol Biomarkers Prev 24:1381

4. Kroh EM, Parkin RK, Mitchell PS, Tewari M (2010) Analysis of circulating microRNA biomarkers in plasma and serum using quantitative reverse transcription-PCR (qRT-PCR). Methods 50:298-301.

**Supplementary Table 1**. Clinical features of older adults with high or low circulating miR-125b levels

|  | **Total**  **(n = 384)** | **High**  **(n = 186)** | **Low (n = 198)** | ***p-value*** |
| --- | --- | --- | --- | --- |
|  |
| *Demographic profile* |  |  |  |  |
| **Age (years)** | 74.1 ± 6.2 | 73.9 ± 6.2 | 74.3 ± 6.2 | *0.604* |
| **Sex (male %)** | 152 (39.6) | 84 (45.2) | 68 (34.3) | *0.030** |
| *Lifestyle factors* |  |  |  |  |
| **Smoking (%)** | 12 (3.1) | 5 (2.7) | 7 (3.5) | *0.635* |
| **Alcohol consumption (%)** | 85 (22.1) | 47 (25.3) | 38 (19.2) | *0.153* |
| **Regular exercise (%)** | 328 (85.4) | 162 (87.1) | 166 (83.8) | *0.367* |
| *Comorbidities* |  |  |  |  |
| **Hypertension (%)** | 166 (43.2) | 84 (45.2) | 82 (41.4) | *0.460* |
| **Diabetic mellitus (%)** | 42 (10.9) | 13 (7.0) | 29 (14.7) | *0.016** |
| **Hyperlipidemia (%)** | 75 (19.5) | 37 (19.9) | 38 (19.2) | *0.863* |
| **Coronary artery disease (%)** | 72 (18.8) | 39 (21.0) | 33 (16.7) | *0.282* |
| **Prior cerebrovascular disease (%)** | 10 (2.6) | 5 (2.7) | 5 (2.5) | *0.920* |
| **Gout (%)** | 19 (5.0) | 12 (6.5) | 7 (3.5) | *0.189* |
| **Peptic ulcer (%)** | 68 (17.7) | 36 (19.4) | 32 (16.2) | *0.414* |
| **Chronic liver disease (%)** | 29 (7.6) | 12 (6.5) | 17 (8.6) | *0.430* |
| **Chronic kidney disease (%)** | 12 (3.1) | 6 (3.2) | 6 (3.0) | *0.913* |
| **Prostatic hyperplasia (%)** | 73 (19.0) | 35 (18.8) | 38 (19.2) | *0.926* |
| **Thyroid disorder (%)** | 55 (14.3) | 31 (16.7) | 24 (12.1) | *0.205* |
| **Chronic obstructive pulmonary disease (%)** | 23 (6.0) | 13 (7.0) | 10 (5.1) | *0.425* |
| **Malignancy (%)** | 19 (5.0) | 11 (5.9) | 8 (4.0) | *0.399* |
| *Regular medications* |  |  |  |  |
| **Anti-hypertension medications (%)** | 159 (41.4) | 81 (43.6) | 78 (39.4) | *0.410* |
| **Anti-diabetic medications (%)** | 39 (10.2) | 12 (6.5) | 27 (13.6) | *0.020** |
| **Anti-hyperlipidemic medications (%)** | 58 (15.1) | 30 (16.1) | 28 (14.1) | *0.588* |
| *Physical parameters* |  |  |  |  |
| **Systolic blood pressure (mmHg)** | 129.5 ± 16.8 | 130.5 ± 15.0 | 128.5 ± 18.2 | *0.237* |
| **Diastolic blood pressure (mmHg)** | 74.4 ± 9.8 | 75.3 ± 9.4 | 73.7 ± 10.1 | *0.112* |
| **Heart rate (/min)** | 70.4 ± 10.6 | 70.3 ± 10.4 | 70.5 ± 10.9 | *0.896* |
| **Body height (cm)** | 158.3 ± 7.9 | 159.1 ± 7.9 | 157.5 ± 7.8 | *0.041* |
| **Body weight (kg)** | 59.5 ± 10.6 | 60.6 ± 11.1 | 58.4 ± 10.0 | *0.048* |
| **Body mass index (kg/m^2^)** | 24.2 ± 10.3 | 24.9 ± 14.4 | 23.5 ± 3.3 | *0.197* |
| **Waste circumference (cm)** | 81.5 ± 9.4 | 82.4 ± 9.6 | 80.5 ± 9.2 | *0.049* |
| *Hemogram* |  |  |  |  |
| **Leukocyte (K/μL)** | 5.4 ± 1.5 | 5.4 ± 1.4 | 5.3 ± 1.5 | *0.430* |
| **Platelet (K/μL)** | 214.5 ± 58.6 | 215.8 ± 59.1 | 213.3 ± 58.2 | *0.677* |
| **Hemoglobin (g/dL)** | 13.7 ± 1.3 | 13.9 ± 1.3 | 13.5 ± 1.2 | *0.012* |
| **Red-cell distribution width** | 13.2 ± 0.9 | 13.1 ± 0.9 | 13.3 ± 0.9 | *0.037* |
| *Serum biochemistry* |  |  |  |  |
| **Albumin (mg/dL)** | 4.3 ± 0.2 | 4.3 ± 0.3 | 4.3 ± 0.2 | *0.795* |
| **Globulin (mg/dL)** | 2.8 ± 0.3 | 2.7 ± 0.3 | 2.8 ± 0.4 | *0.173* |
| **A/G ratio** | 1.6 ± 0.2 | 1.6 ± 0.2 | 1.6 ± 0.2 | *0.685* |
| **Urea nitrogen (mg/dL)** | 16.8 ± 5.1 | 17.3 ± 5.3 | 16.3 ± 4.9 | *0.059* |
| **Creatinine (mg/dL)** | 0.8 ± 0.3 | 0.8 ± 0.3 | 0.8 ± 0.3 | *0.122* |
| **eGFR (mL/min/1.73 m^2^)** | 90.7 ± 22.4 | 89.6 ± 23.6 | 91.7 ± 21.2 | *0.348* |
| **Glucose (mg/dL)** | 99.1 ± 17.2 | 98.1 ± 16.7 | 100.0 ± 17.7 | *0.275* |
| **Uric acid (mg/dL)** | 5.5 ± 1.2 | 5.6 ± 1.2 | 5.5 ± 1.2 | *0.173* |
| **Total cholesterol (mg/dL)** | 186.7 ± 31.4 | 184.3 ± 32.0 | 189.1 ± 30.7 | *0.136* |
| **Triglyceride (mg/dL)** | 111.5 ± 64.1 | 115.1 ± 69.2 | 108.1 ± 58.8 | *0.288* |
| **Low density lipoprotein cholesterol (mg/dL)** | 108.1 ± 26.5 | 107.0 ± 27.2 | 109.2 ± 25.9 | *0.432* |
| **High density lipoprotein cholesterol (mg/dL)** | 56.8 ± 14.3 | 55.9 ± 14.6 | 57.6 ± 14.0 | *0.225* |

*eGFR, estimated glomerular filtration rate based on the chronic kidney disease – epidemiology collaboration (CKD-EPI) formula*

** p < 0.05*

**Supplementary Table 2**. Log-transformed circulating miR-125b levels according to categorical variables

|  | **Log-transformed miR-125b levels** | | ***p-value*** |
| --- | --- | --- | --- |
| *Demographic profile* | *Male* | *Female* |  |
| **Sex** | -1.91 ± -0.87 | -1.97 ± -0.8 | *0.481* |
| *Lifestyle factors* | *With* | *Without* |  |
| **Smoking** | -2.03 ± -0.83 | -1.95 ± -0.83 | *0.723* |
| **Alcohol consumption** | -1.94 ± -0.83 | -1.95 ± -0.83 | *0.879* |
| **Regular exercise** | -1.94 ± -0.82 | -2.02 ± -0.86 | *0.459* |
| *Comorbidities* | *With* | *Without* |  |
| **Hypertension** | -1.89 ± -0.83 | -1.99 ± -0.83 | *0.266* |
| **Diabetic mellitus** | -2.30 ± -0.79 | -1.90 ± -0.82 | *0.003** |
| **Hyperlipidemia** | -1.91 ± -0.76 | -1.96 ± -0.85 | *0.654* |
| **Coronary artery disease** | -1.88 ± -1.03 | -1.96 ± -0.77 | *0.440* |
| **Prior cerebrovascular disease** | -1.92 ± -1.08 | -1.95 ± -0.82 | *0.901* |
| **Gout** | -1.85 ± -0.74 | -1.95 ± -0.83 | *0.606* |
| **Peptic ulcer** | -1.90 ± -0.96 | -1.96 ± -0.80 | *0.621* |
| **Chronic liver disease** | -2.08 ± -0.82 | -1.94 ± -0.83 | *0.381* |
| **Chronic kidney disease** | -1.85 ± -1.07 | -1.95 ± -0.82 | *0.666* |
| **Prostatic hyperplasia** | -1.97 ± -0.97 | -1.94 ± -0.79 | *0.784* |
| **Thyroid disorder** | -1.86 ± -0.91 | -1.96 ± -0.81 | *0.418* |
| **Chronic obstructive pulmonary disease** | -1.76 ± -0.79 | -1.96 ± -0.83 | *0.271* |
| **Malignancy** | -1.75 ± -0.63 | -1.96 ± -0.84 | *0.279* |
| *Medications* | *With* | *Without* |  |
| **Anti-hypertension medications** | -1.87 ± -0.82 | -2.00 ± -0.83 | *0.137* |
| **Anti-diabetic medications** | -2.32 ± -0.81 | -1.91 ± -0.82 | *0.003** |
| **Anti-hyperlipidemic medications** | -1.91 ± -0.76 | -1.95 ± -0.84 | *0.708* |

** p < 0.05*

**Supplementary Table 3**. Original miR-125b-5p levels according to categorical variables

|  | **miR-125b-5p levels** | | ***p-value*** |
| --- | --- | --- | --- |
| *Demographic profile* | *Male* | *Female* |  |
| **Sex** | 0.0155 (0.0033, 0.0408) | 0.0102 (0.003, 0.0369) | *0.324* |
| *Lifestyle factors* | *With* | *Without* |  |
| **Smoking** | 0.0069 (0.0028, 0.0297) | 0.0117 (0.0031, 0.04) | *0.700* |
| **Alcohol consumption** | 0.0136 (0.0032, 0.0335) | 0.011 (0.0031, 0.0433) | *0.791* |
| **Regular exercise** | 0.0118 (0.0031, 0.0428) | 0.011 (0.0033, 0.0318) | *0.354* |
| *Comorbidities* | *With* | *Without* |  |
| **Hypertension** | 0.0122 (0.0037, 0.0411) | 0.01 (0.0028, 0.0376) | *0.318* |
| **Diabetic mellitus** | 0.0051 (0.002, 0.0153) | 0.0122 (0.0033, 0.0451) | *0.002* |
| **Hyperlipidemia** | 0.0117 (0.0035, 0.0291) | 0.0114 (0.0029, 0.0403) | *0.837* |
| **Coronary artery disease** | 0.0175 (0.0024, 0.077) | 0.0112 (0.0033, 0.0369) | *0.486* |
| **Prior cerebrovascular disease** | 0.0108 (0.0019, 0.0691) | 0.0116 (0.0032, 0.0388) | *0.885* |
| **Gout** | 0.0219 (0.006, 0.0566) | 0.0113 (0.003, 0.038) | *0.410* |
| **Peptic ulcer** | 0.0147 (0.0029, 0.053) | 0.0112 (0.0032, 0.036) | *0.722* |
| **Chronic liver disease** | 0.0087 (0.0021, 0.0222) | 0.0118 (0.0032, 0.0403) | *0.292* |
| **Chronic kidney disease** | 0.0132 (0.002, 0.0415) | 0.0116 (0.0031, 0.0392) | *0.928* |
| **Prostatic hyperplasia** | 0.0093 (0.0029, 0.0407) | 0.0117 (0.0032, 0.0387) | *0.711* |
| **Thyroid disorder** | 0.0134 (0.0028, 0.0905) | 0.0112 (0.0031, 0.0359) | *0.378* |
| **Chronic obstructive pulmonary disease** | 0.0185 (0.0037, 0.0978) | 0.0114 (0.003, 0.0372) | *0.290* |
| **Malignancy** | 0.0151 (0.0078, 0.0445) | 0.0114 (0.0029, 0.039) | *0.205* |
| *Medications* | *With* | *Without* |  |
| **Anti-hypertension medications** | 0.0122 (0.0038, 0.0413) | 0.0099 (0.0027, 0.0372) | *0.177* |
| **Anti-diabetic medications** | 0.0049 (0.0019, 0.015) | 0.0122 (0.0033, 0.0444) | *0.002* |
| **Anti-hyperlipidemic medications** | 0.0136 (0.0035, 0.0379) | 0.0113 (0.003, 0.0396) | *0.751* |

**Supplementary Table 4**. Multiple logistic regression analyses with having high circulating miR-125b as the dependent variable

|  | **Odds ratio** | **95% CI** | ***p-value*** |
| --- | --- | --- | --- |
| *Model 1: included clinical and physical parameters^1^* | |  |  |
| Diabetes mellitus | 0.362 | 0.177 – 0.738 | *0.005* |
| Waist circumference (cm) | 1.029 | 1.006 – 1.052 | *0.013* |
| *Model 2: model 1 variables + laboratory data^2^* | |  |  |
| Diabetes mellitus | 0.355 | 0.173 – 0.728 | *0.005* |
| Waist circumference (cm) | 1.029 | 1.006 – 1.052 | *0.013* |
| RDW | 0.78 | 0.617 – 0.986 | *0.038* |

*CI, confidence interval; RDW, red cell distribution width*

^1^ Including age, gender, diabetes mellitus, use of anti-diabetic medications, body height, body weight, waist circumference

**^2^** Including age, gender, diabetes mellitus, use of anti-diabetic medications, body height, body weight, waist circumference, hemoblogin, and RDW
